# miR-124 inhibits proliferation and invasion of human retinoblastoma cells by targeting STAT3

**DOI:** 10.3892/or.2021.8193

**Published:** 2021-09-23

**Authors:** Shu Liu, Chunmei Hu, Yingxue Wang, Guang Shi, Yarong Li, Huang Wu

Oncol Rep 36: 2398-2404, 2016; DOI: 10.3892/or.2016.4999

Following the publication of this paper, it was drawn to the Editors attention by a concerned reader that the GAPDH bands shown in the western blots in Fig. 6A were strikingly similar to data appearing in different form in other articles by different authors. Owing to the fact that the contentious data in the above article had already been published elsewhere prior to its submission to *Oncology Reports*, the Editor has decided that this paper should be retracted from the Journal. After having been in contact with the authors, they agreed with the decision to retract the paper. The Editor apologizes to the readership for any inconvenience caused.

